# A contribution-interpretable belief rule base model for obesity risk prediction using physical indicators and dietary habits

**DOI:** 10.3389/fendo.2025.1706780

**Published:** 2025-12-08

**Authors:** Fanxu Wei, Li Jiang, Hailong Zhu, Yinling Mao

**Affiliations:** 1 School of Computer Science and Information Engineering, Harbin Normal University, Harbin, China; 2 Department of Hematology, Harbin Medical University Cancer Hospital, Harbin, China; 3 First Ward of Abdominal Radiotherapy, Harbin Medical University Cancer Hospital, Harbin, China

**Keywords:** obesity prediction, dietary behaviors, belief rule base, hybrid optimization, lifestyle management

## Abstract

**Introduction:**

Obesity is a pressing global endocrine disorder that disrupts hormonal axes and elevates the risk of type 2 diabetes, cardiovascular disease, and metabolic conditions. Early and reliable risk prediction is crucial for timely intervention, yet most existing models fail to balance predictive accuracy with interpretability, limiting their clinical applicability.

**Methods:**

We propose a dual-layer belief rule base model with hybrid evolutionary optimization and contribution analysis (DBRB-C) for multilevel obesity risk prediction. The framework integrates data-driven learning with knowledge-based reasoning through a hierarchical architecture, employs a differential evolution–particle swarm optimization strategy for parameter learning, and introduces a contribution belief matrix with reverse contribution analysis to trace decision-making pathways.

**Results:**

Evaluated on real-world datasets from Latin America and Turkey, DBRB-C achieved higher accuracy and stability than conventional machine-learning models. The model explicitly identified modifiable dietary behaviors—such as vegetable consumption frequency and number of daily meals—as key determinants of obesity risk, while providing fully traceable reasoning for each prediction.

**Discussion:**

The DBRB-C model successfully bridges the gap between accuracy and interpretability in obesity risk assessment. Its transparent, contribution-aware architecture offers a reliable foundation for personalized lifestyle interventions and supports evidence-based clinical decision-making in endocrinology and preventive health.

## 1 Introduction

Obesity has emerged as a pressing global endocrine-related health challenge, disrupting key hormonal axes (e.g., the insulin–glucose regulatory axis and the leptin–ghrelin axis), and is strongly associated with type 2 diabetes mellitus (T2DM), dyslipidemia, thyroid dysfunction, and polycystic ovary syndrome (PCOS)—conditions that are central to clinical endocrinology (1, 2). For endocrinologists, accurate prediction of obesity severity (e.g., BMI classification, visceral adiposity grading) is pivotal for early risk stratification of hormone-dependent comorbidities and for designing personalized interventions (e.g., lifestyle guidance, insulin sensitizers) (3). Despite advances in predictive modeling, existing approaches often lack transparency in interpreting endocrine-relevant features and in tracing the basis of predictions, limiting their utility in real-world endocrine clinical settings.

Current methodologies can be categorized into data-driven and knowledge-driven approaches, each with specific advantages and limitations. Data-driven methods typically achieve high predictive accuracy but offer limited interpretability and feature contribution tracing capabilities. For instance, Yagin et al. proposed a multilayer perceptron neural network with Bayesian optimization, achieving 93.06% classification accuracy, yet the model provides no mechanism for explaining its predictions (4). Maulana et al. applied CatBoost to health survey data from Mexico, Peru, and Colombia, achieving 95.98% accuracy and identifying key features, but the reasoning process remains opaque (5). Similarly, Delpino et al. reviewed 14 machine learning studies and found that random forests achieved an area under the receiver operating characteristic curve (AUC) of 0.86, but interpretability was limited (6). Overall, data-driven approaches excel in predictive performance but are often “black boxes.”

In contrast, knowledge-driven methods build models based on domain knowledge and medical theory, emphasizing interpretability. Gozukara Bag et al. combined logistic regression with feature selection to analyze the impact of gender, diet, physical activity, and family history, achieving robust accuracy and AUC, but handling complex interactions remained challenging (7). Jeon et al. applied machine learning classifiers to identify age-specific obesity risk factors, yet the reasoning paths were unclear (8). Thamrin et al. analyzed Indonesian health survey data with logistic regression, noting limitations under class imbalance (9). Knowledge-driven approaches thus provide interpretability but often sacrifice predictive precision and scalability.

The growing demand for models that combine high predictive accuracy with contribution tracing and interpretable reasoning has led to hybrid approaches, integrating data-driven learning with knowledge-driven inference. Among these, the belief rule base (BRB) framework has shown promise due to its balance of accuracy and interpretability (10). However, current BRB-based obesity prediction studies face two challenges: i) most use a single-layer classification structure, limiting hierarchical reasoning and resulting in opaque inference paths (11); and ii) forward inference dominates, with limited backward contribution tracing, reducing result traceability.

To address these challenges, we propose DBRB-C. The method aims to achieve accurate prediction while providing transparent reasoning and contribution traceability. Its main contributions are as follows:

1. Dual-layer hierarchical classification: A coarse-to-fine modular framework simplifies rule structures, transforming global complex reasoning into hierarchical, interpretable paths.
2. Hybrid DE–PSO optimization: This integrates global and local search to improve accuracy without sacrificing interpretability; feature weights retain clear physical meaning.
3. Backward contribution reasoning: This introduces a contribution belief matrix (CBM) to quantify features’ relative contributions to obesity classification outcomes via reverse contribution analysis (RCA), ensuring traceable outcomes and supporting clinical interventions.

The remainder of this paper is organized as follows. Section 2 introduces the DBRB-C model and dataset. Section 3 presents the experimental results. Section 4 discusses the implications for obesity assessment and interventions. Section 5 concludes and outlines future directions.

## 2 Materials and methods

This chapter describes the development and evaluation framework of the DBRB-C model for multilevel obesity prediction and interpretable contribution analysis. Section 2.1 introduces the dataset, Section 2.2 presents model development, Section 2.3 details the data partitioning and evaluation, and Section 2.4 outlines the parameter settings and optimization strategy.

### 2.1 Dataset

This study first adopted the dataset compiled and published by Palechor and Manotas, which was designed to assess individual obesity levels based on dietary behaviors and physical conditions. It included samples from Colombia, Peru, and Mexico, with respondents aged between 14 and 61 years old. A total of 2,111 records were collected through online questionnaires. The dataset covered basic demographic information, dietary habits, lifestyle, and physical activity data, including 17 variables, and all samples were labeled with standardized obesity-level labels.

Specifically, the 17 features of this dataset include gender, age, height, weight, frequency of high-calorie food consumption (FAVC), frequency of vegetable consumption (FCVC), number of main meals per day (NCP), snacking behavior (CAEC), daily water intake (CH_2_O), frequency of alcohol consumption (CALC), calorie intake monitoring (SCC), frequency of physical activity (FAF), electronic device usage time (TUE), transportation mode (MTRANS), smoking status, and family history of overweight.

**Figure 1:**
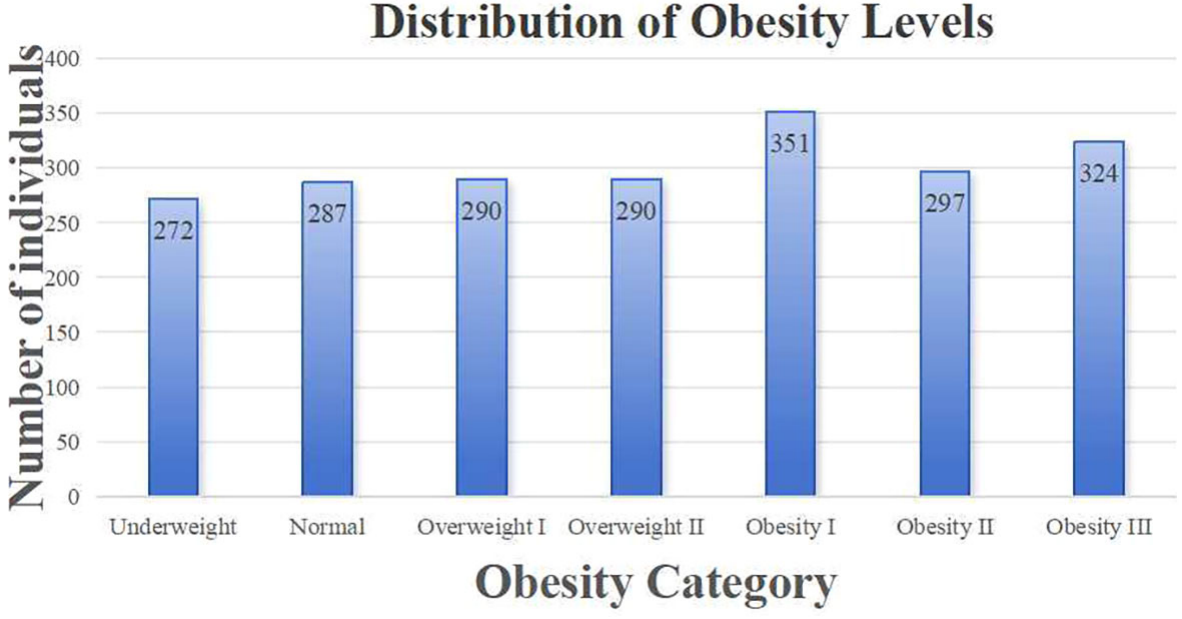
Distribution of obesity levels.

The obesity-level labels of this dataset were defined in accordance with the body mass index (BMI) classification system proposed by the World Health Organization (WHO) and the Mexican National Standard (Norma Oficial Mexicana, NOM-008-SSA3-2010). The specific criteria are presented in **Table 1**, and the detailed data distribution is shown in **Figure 1**.

**Table 1:** BMI classification criteria for obesity levels.

Obesity levels	BMI range (kg/m²)
Insufficient weight	<18.5
Normal weight	18.5∼24.9
Overweight level I	25.0∼29.9
Obesity type I	30.0∼34.9
Obesity type II	35.0∼39.9
Obesity type III	≥40.0

To improve data representativeness and address the generalization limitation potentially caused by single-region samples, the other dataset used in this paper is a supplementary obesity dataset constructed through an online questionnaire survey among residents in Türkiye. The respondents were aged between 18 and 54 years old, covering a typical adult population. A total of 1,610 records were collected, including 14 variables related to obesity assessment. The obesity-level labeling of this Turkish dataset strictly follows the core framework of the WHO adult BMI classification system, ensuring that the classification conforms to the basic definition of weight health status in the public health field, and this classification standard is consistent with the WHO classification system followed by the first dataset.

The 14 specific features of this dataset include gender, age, height, family history of overweight/obesity, fast food consumption, frequency of vegetable intake, number of main meals per day, intermeal snacking behavior, daily liquid intake, frequency of physical exercise, technology product usage time, transportation mode, smoking status, and habit of calculating calorie intake.

### 2.2 Model development

First, the basic framework of the DBRB-C model includes a structured reasoning framework and a result traceability framework. The structured reasoning framework is designed based on the RIMER method. The belief rule can be formally expressed as:


$$\begin{array}{c} \mathrm{IF}\left(x_{1}\;is\;A_{1}^{k}\right)\wedge \left(x_{2}\;is\;A_{2}^{k}\right)\wedge \ldots \wedge \left(x_{T_{k}}\;is\;A_{T_{k}}^{k}\right)\\ \mathrm{THEN}\;y=\left\{\left(D_{1},\beta_{1}^{k}\right),\left(D_{2},\beta_{2}^{k}\right),\ldots ,\left(D_{N},\beta_{N}^{k}\right)\right\},\;\mathrm{subject}\;to\;\sum_{i=1}^{N}\beta_{1}^{k}\leq 1,\\ \mathrm{with}\;\theta_{k}\left(k=1,2,\ldots ,L\right)\;\mathrm{and}\;\delta_{i}\left(i=1,2,\ldots ,T_{k}\right). \end{array}$$

**Figure 2:**
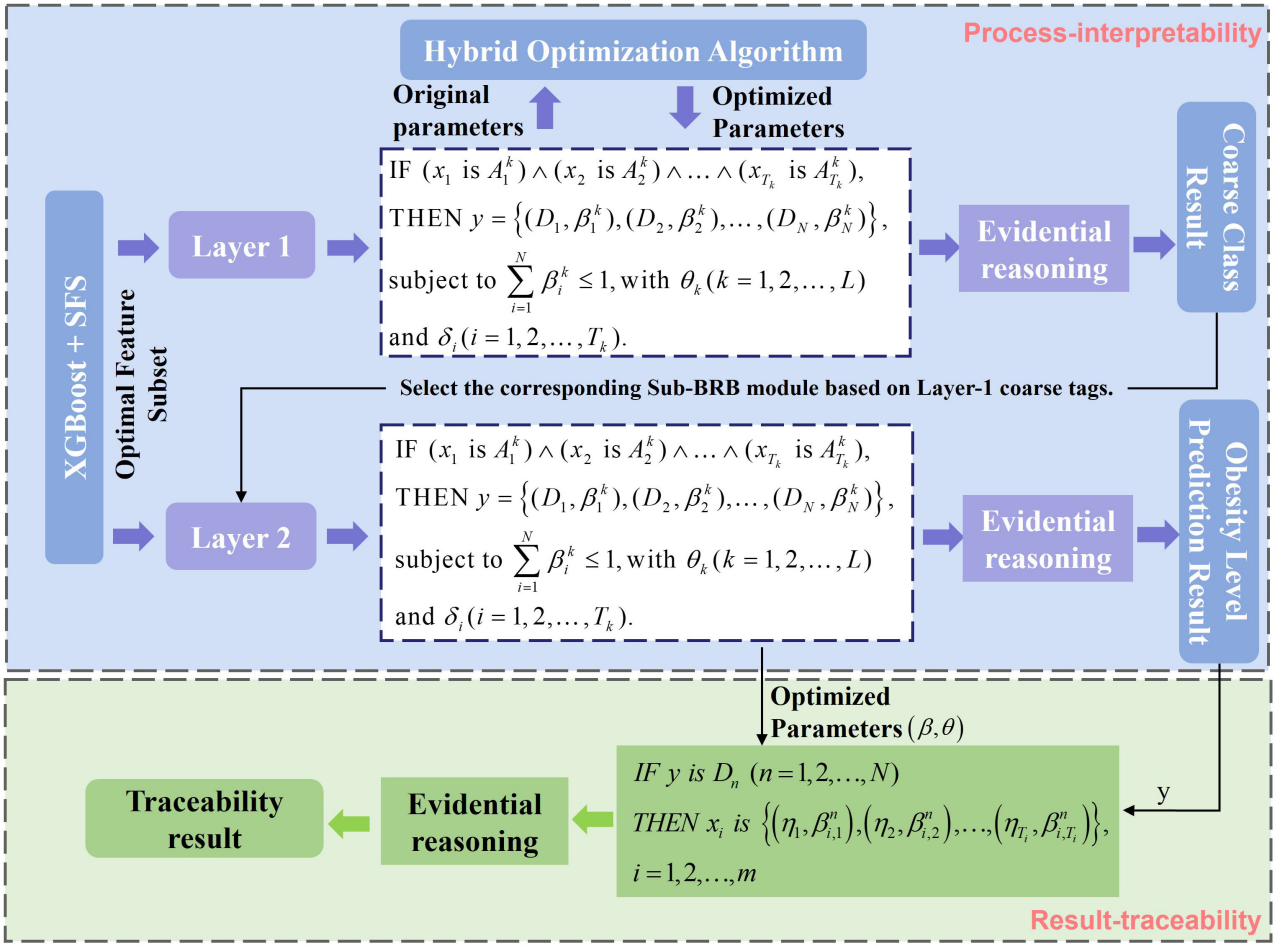
The DBRB-C model for obesity level prediction.

Here, $x_{i}\left(i=1,2,\ldots ,T_{k}\right)$ denotes the premise attribute of the rule , $A_{j}^{k}\left(j=1,2,\ldots ,T_{k}\right)$ represents the reference value of the *j* attribute, $\beta_{i}^{k}\left(j=1,2,\ldots ,N\right)$ indicates the belief degree of output level $D_{i}$, $\theta_{k}$ denotes the weight of the *i*-th rule, $\delta_{i}$ represents the weight of the *i* attribute, *L* indicates the total number of rules, and $T_{k}$ denotes the number of premise attributes in the rule . The overall modeling framework is shown in **Figure 2**.

DBRB-C was developed to predict multilevel obesity and provide interpretable insights into feature contributions. To ensure model efficiency and clinical relevance, a two-stage feature selection strategy was applied. First, XGBoost evaluated the global importance of each feature, allowing the exclusion of low-contribution variables (10). Second, sequential forward selection (SFS) iteratively added features that improved prediction accuracy on a validation set (11). This combination reduces complexity while maintaining high predictive performance and interpretability.

**Figure 3:**
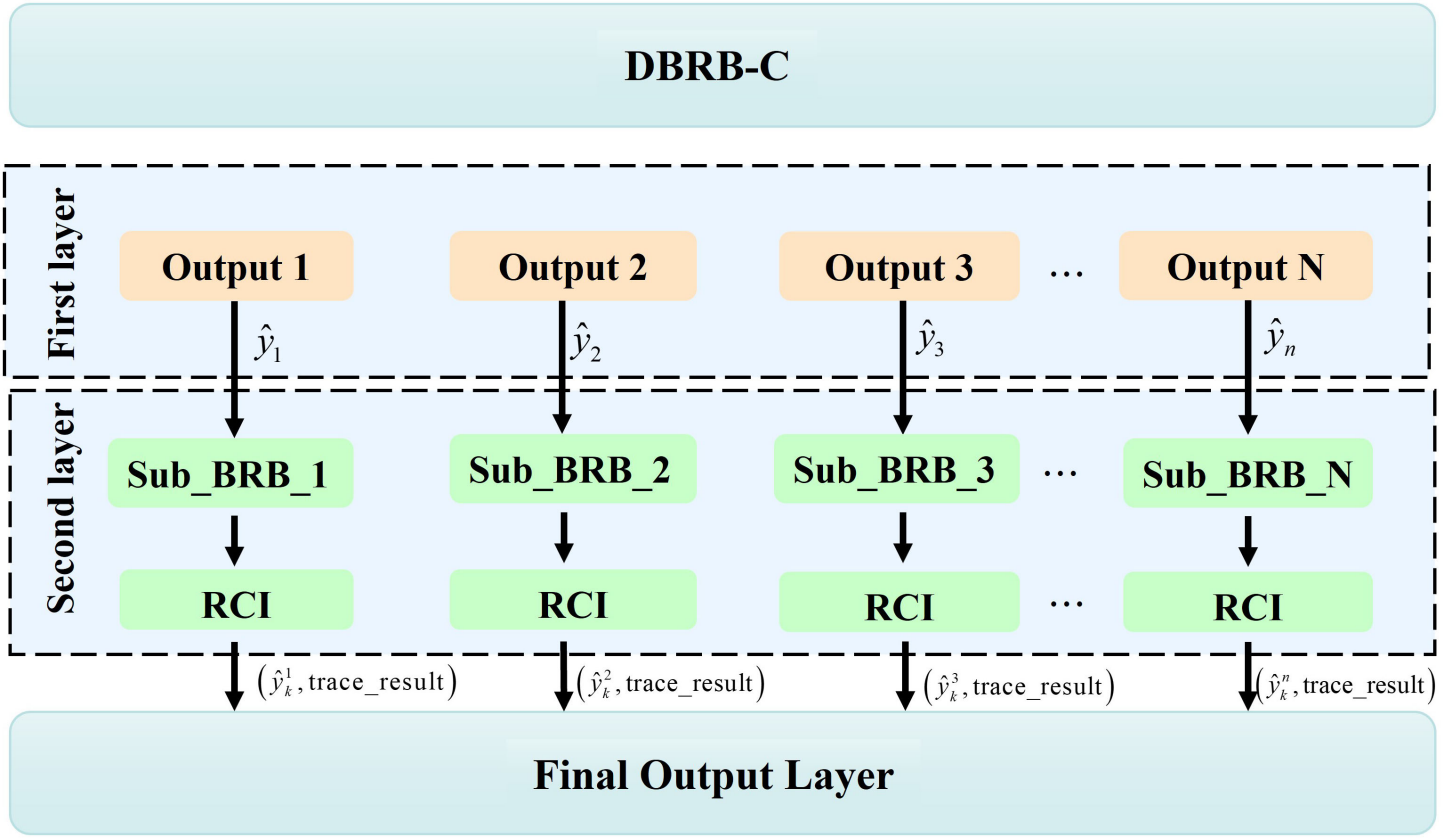
Hierarchical structure of DBRB-C.

As shown in **Figure 3**, to effectively address the challenges in obesity-level prediction, such as complex multiclass label structures and exponential growth in the number of rules, a modular two-layer hierarchical structure was designed in the DBRB-C model. The structure included two stages: a coarse classification layer and a fine classification layer (12). The coarse classification layer separates individuals into lean, overweight, and obese groups, providing a broad categorization of obesity severity. This layer informs the subsequent fine classification layer, which refines the coarse labels to capture subtle differences within each group. Each submodule incorporates the most relevant features, ensuring computational efficiency and clear reasoning paths. This hierarchical design addresses challenges associated with multiclass classification and exponential rule growth while preserving interpretability (13).

**Figure 4:**
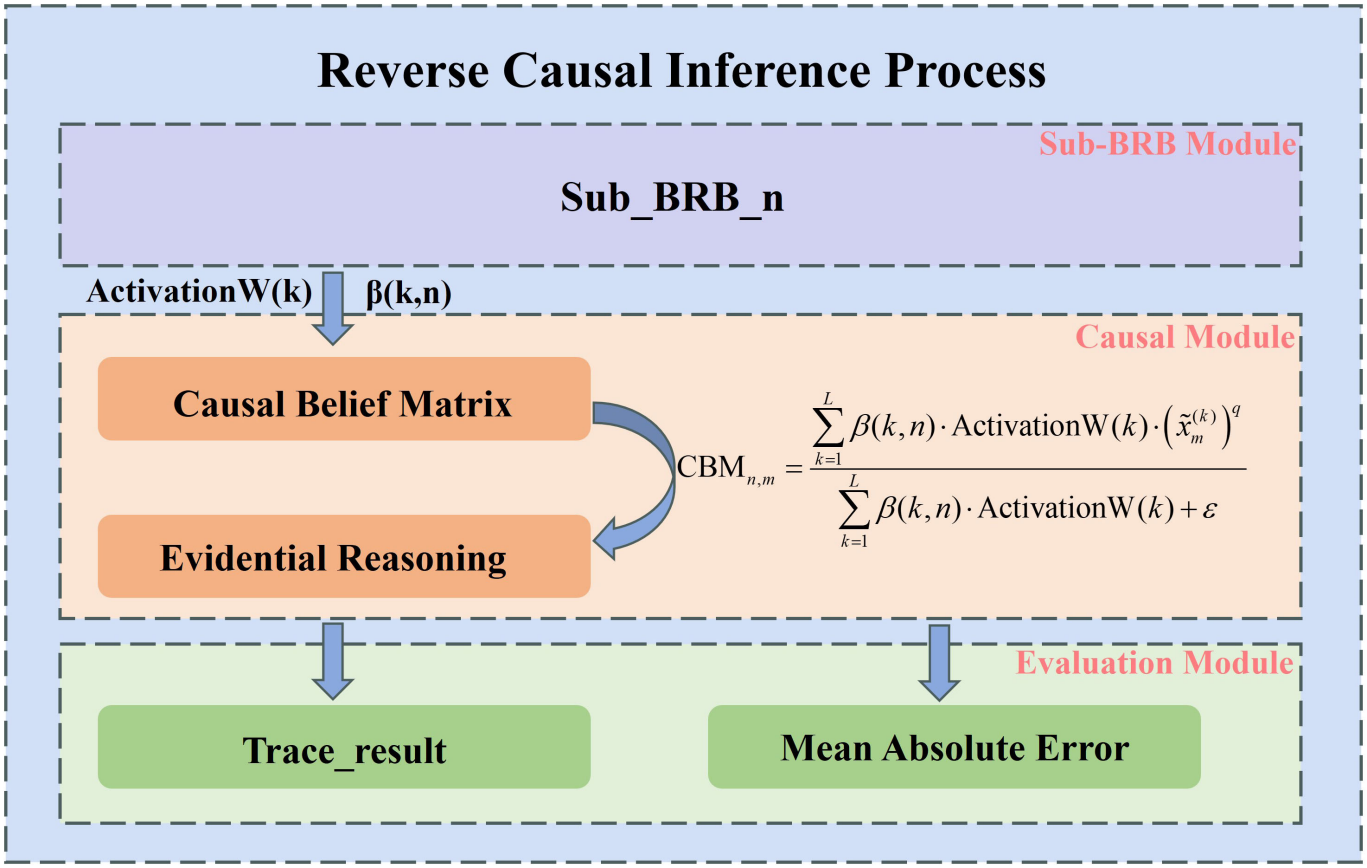
The RCA process in the DBRB-C model.

The DBRB-C model uses the evidential reasoning (ER) algorithm as its core inference mechanism. The inference process consists of two stages: forward inference for predicting obesity levels and RCA for tracing the contribution of input attributes to the predictions (14). Forward inference synthesizes information from multiple features to generate probabilistic predictions of obesity levels. As shown in **Figure 4**, to enhance the interpretability of the model outputs, an RCA mechanism was introduced after the completion of forward inference. A CBM was constructed to quantify the relative contributions of input attributes to the predicted results. This mechanism not only improved the transparency of the model but also provided a quantitative reference for clinical interventions (15). By integrating forward and reverse reasoning, the model not only provides accurate predictions but also highlights the key physiological and lifestyle factors driving these predictions, enhancing interpretability and clinical utility.

**Figure 5:**
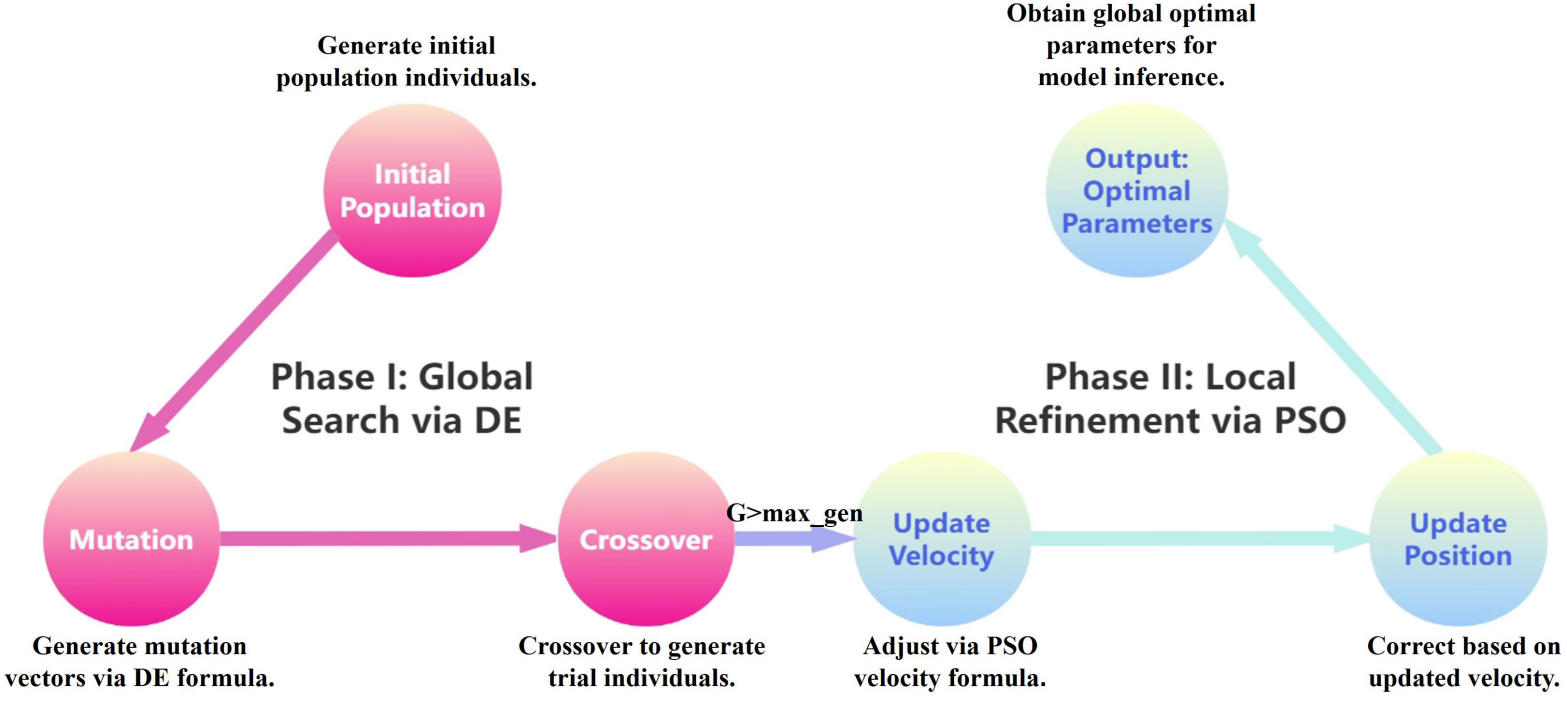
DE-PSO optimization process.

To avoid local optima and to improve both parameter optimization accuracy and model convergence efficiency, differential evolution (DE) and particle swarm optimization (PSO) were combined in a cascaded manner, forming a two-stage joint optimization strategy (16). In this strategy, the strong global search capability of DE was first applied to rapidly explore the global optimal region. Then, the local search advantage of PSO was utilized for fine-tuning (17). The complete workflow is illustrated in **Figure 5**.

Model parameters were optimized using a two-stage hybrid approach combining DE and PSO. DE performs a global search to identify promising parameter regions, and PSO refines these regions through local optimization. This hybrid strategy improves convergence efficiency and model stability, ensuring consistent predictive performance across all obesity categories. **Table 2** summarizes the key structural and optimization parameter settings.

**Table 2:** Parameter configuration of the DBRB-C model.

Parameter	Value	Description
M	Varies	Number of input attributes
R	3	Number of reference values
*L*	*R^M^*	Number of rules
NP	50	DE population size
F	0.6	DE scaling factor
CR	0.9	Crossover rate in DE
w	0.7	Inertia weight in PSO
*C*_1_, *C*_2_	1.5	Learning factors
G	200	Maximum iterations
α	0.7	Fitness weight

### 2.3 Data partitioning and evaluation

To ensure the scientific validity of model training and the verifiability of inference performance, the original dataset was divided into training, validation, and test sets at a ratio of 6:2:2. These sets were used for parameter learning, structural tuning, and final performance evaluation, respectively. This partitioning scheme is widely adopted in supervised learning and effectively balances training efficiency and generalization capability (18).

The original dataset was partitioned at the 6:2:2 ratio via stratified sampling, with the specific implementation process as follows: First, the sample indices corresponding to each label were screened and separated one by one to ensure independent partitioning of samples in each category. Second, for the sample pool of each label, the randperm function was used to randomly shuffle the sample indices, eliminating the selection bias that might be introduced by the original data ordering. Third, based on the preset 6:2:2 ratio, the number of samples allocated to the training, validation, and test sets under each label was calculated. Finally, according to the calculated allocation quantity, the corresponding samples were extracted from the shuffled sample pools of each label, and the samples of all labels were merged to construct the final training, validation, and test sets for model training, tuning, and evaluation. This stratified sampling method ensures that the proportion of samples of each obesity level in the training, validation, and test sets is completely consistent with that in the original dataset, effectively avoiding the impact of class imbalance on model training bias and evaluation accuracy. Meanwhile, the fixed random seed and clear step-by-step operation process further improve the reproducibility of the data partitioning process, providing a guarantee for the reliability of subsequent experimental results.

**Figure 6:**
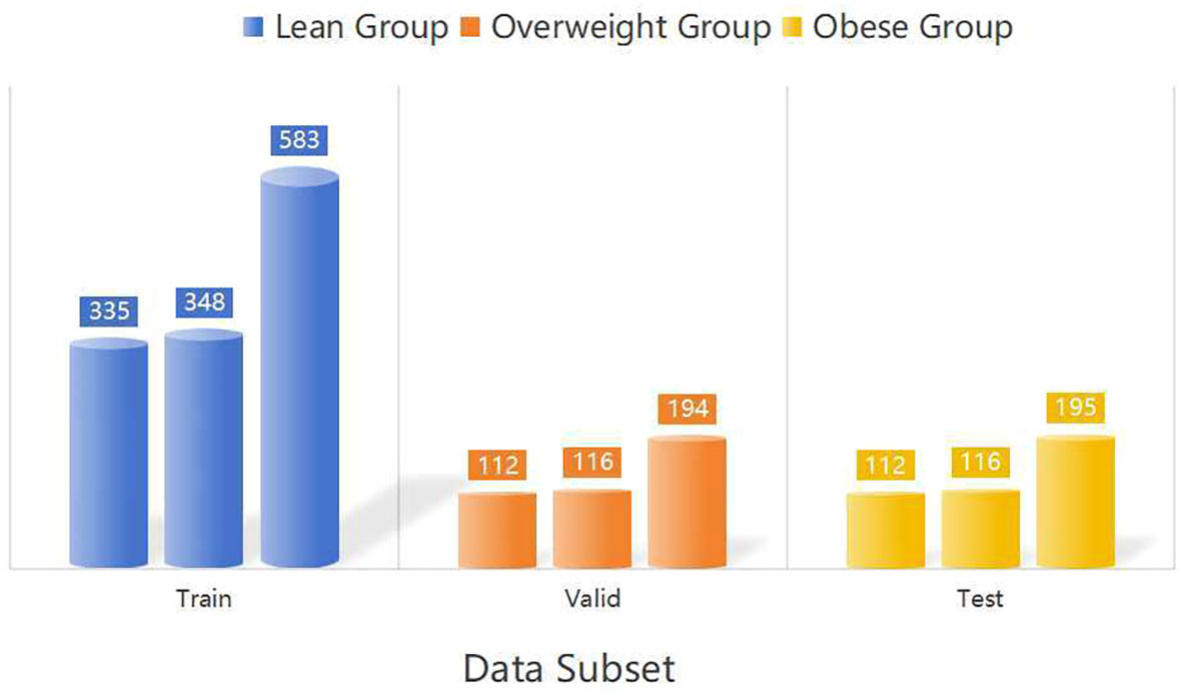
Class distribution in training, validation, and test subsets.

In addition, to ensure that each subset was representative in terms of label distribution, a stratified sampling strategy was applied in the first layer of the model. This ensured that the proportions of the three coarse-grained labels—lean group, overweight group, and obese group—in the training, validation, and test sets remained consistent with the original dataset. This approach prevented class imbalance from affecting model performance (19). Detailed data distribution is shown in **Figure 6**.

It should be noted that the three coarse-grained labels were derived by merging the seven fine-grained labels according to the BMI classification standards proposed by the WHO and National Institutes of Health (NIH). The specific merging scheme is presented in **Table 3**. Overall, the three-way split into training, validation, and test sets not only ensured the scientific rigor of the model tuning process but also provided a systematic basis for performance evaluation.

**Table 3:** Coarse-level BMI grouping.

Group name	BMI range (kg/m²)	Included categories
Lean group	BMI < 25	Insufficient weight, normal weight
Overweight group	25.0 ≤ BMI < 29.9	Overweight level I, II
Obese group	BMI ≥ 30.0	Obesity I, II, III

Performance evaluation was conducted using both standard classification metrics and error-based measures. Standard metrics, including accuracy (20), precision (21), recall (22), and F1-score (23), provided a comprehensive assessment of the model’s recognition ability across categories, as well as sensitivity and balance between precision and recall.

In addition, error-based indicators were employed to evaluate model reliability from a different perspective. The mean absolute error (MAE) quantified the deviation between predicted and true values, reflecting both forward prediction accuracy and the validity of RCA (24). A normalized MAE was also introduced to account for attribute range heterogeneity, providing a quantitative measure for the credibility of backward inference results (25).

### 2.4 Parameter settings

To balance the inference accuracy and interpretability of the DBRB-C model, both structural parameters and optimization hyperparameters were systematically defined. The parameter design considered both representational capacity and control of model complexity (26).

In the model, the input feature dimension was determined by the number of selected optimal features. The number of reference values for each attribute defined the belief rules, which allowed sufficient discretization of the input space. At the same time, interpretability and computational tractability were preserved (27). Attribute and rule weight mechanisms were further introduced to handle incomplete information and to enhance flexibility (28).

For the optimization module, a combined DE–PSO strategy was employed. In the DE phase, the population size, scaling factor, and crossover probability were set. In the PSO phase, the inertia factor, learning factors, and maximum iterations were specified. These configurations were used to optimize parameters and to improve convergence efficiency and stability (29). The key parameter settings of the model are summarized in **Table 3** to enhance readability and reproducibility (30).

## 3 Results

This chapter presents the experimental findings of the DBRB-C model for multilevel obesity classification. All results are presented based on the dataset, evaluation metrics, and experimental setup described in Section 2.

### 3.1 Feature selection

A joint feature selection strategy integrating XGBoost and SFS was employed to reduce input dimensionality while preserving the model’s discriminative power, thereby mitigating computational cost and rule explosion caused by redundant features.

During the coarse classification stage, the objective was to distinguish among the lean, overweight, and obese groups (corresponding to class labels 0, 1, and 2). Initially, all input features were evaluated using the gain metric from XGBoost, and features exceeding a threshold of 0.06 were retained. Subsequently, the SFS method was applied to iteratively construct the optimal feature subset based on classification accuracy.

In the fine classification stage, samples are divided into three submodules according to the results of coarse classification, with each submodule addressing a local classification task at a specific level.

Submodule A: Samples with the coarse label of “normal weight group” are further divided into insufficient weight and normal weight.

Submodule B: Samples with the coarse label of “over group” are subdivided into overweight I and overweight II.

Submodule C: Samples with the coarse label of “obesity group” are further divided into obesity I, obesity II, and obesity III.

**Table 4** summarizes the final feature subsets selected for each submodule, reflecting the outcome of the combined XGBoost and SFS selection strategy.

**Table 4:** Final selected feature subsets for each module.

Module name	Target labels	Final selected feature subset
Coarse classification	0, 1, 2	Height, weight
Submodule A	0, 1	Height, weight, NCP
Submodule B	2, 3	Age, height, weight
Submodule C	4, 5, 6	Gender, height, weight, FCVC

To further quantify and validate the effectiveness of the joint feature selection strategy and hierarchical architecture in mitigating the rule explosion problem, a comparison was conducted between the traditional BRB model, which relies solely on XGBoost for feature selection, and the DBRB-C model in terms of rule quantity. This comparison not only demonstrates the advantage of the hierarchical submodules and feature selection strategy in controlling the number of rules but also indicates that the model achieves higher computational efficiency and inference scalability while maintaining high classification performance.

The traditional BRB model used XGBoost to select a feature set suitable for the seven target labels. An important threshold of 0.06 was applied, resulting in six selected features. If three reference values are assigned to each feature, the comparison of rule quantities is presented in **Table 5**.

**Table 5:** Comparison of rule numbers.

Model	Number of features	Number of rules
BRB	6	3^6^=729
DBRB-C	Coarse classifier module: 2	3^2^+3^3^+3^3^+3^4^=144
	Submodule A: 3	
	Submodule B: 3	
	Submodule C: 4	

### 3.2 Module performance

**Table 6** lists the DBRB-C model’s performance metrics for each fine-classification sublabel, including precision, recall, and F1 score for the seven obesity levels. Results show the model achieved high accuracy and balance across all categories.

**Table 6:** Performance of the fine-grained classifiers.

Class label	Precision	Recall	F1 score
Insufficient	100%	98.04%	99.01%
Normal	98.39%	100%	99.19%
Overweight I	96.77%	100%	98.36%
Overweight II	100%	96.43%	98.18%
Obesity I	98.53%	100%	99.26%
Obesity II	98.41%	96.88%	97.64%
Obesity III	98.44%	98.44%	98.44%
Average	98.65%	98.54%	98.58%
Overall accuracy	98.58%		

**Figure 7:**
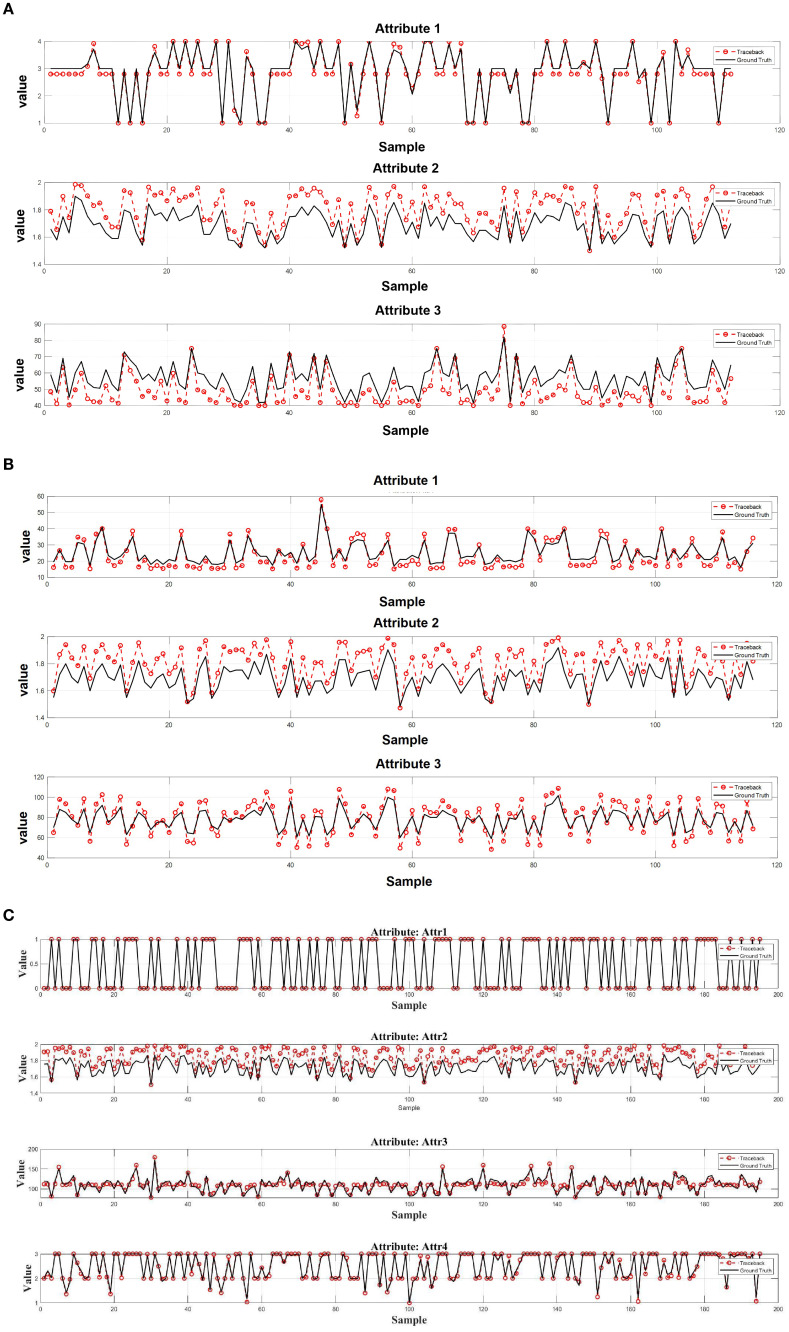
RCA results showing the agreement between reconstructed and true attribute values for each submodule. **(A)** Submodule A (Height, Weight, NCP). **(B)** Submodule B (Age, Height, Weight). **(C)** Submodule C (Gender, Height, Weight, FCVC).

To enhance the model’s traceability and medical validity in multilevel obesity classification, we introduced the RCA method into three fine-grained submodules. **Figure 7** compares each submodule’s backward inference results with observed values—for most samples, predicted attribute values match actual values closely, confirming the model’s accuracy in reconstructing input features.

We calculated the MAE of key attributes to quantify this accuracy. From **Table 7**, meal frequency (NCP, 0.0509) and vegetable consumption (FCVC, 0.0198) have the lowest MAE values, showing the model can accurately reconstruct these dietary behaviors via RCA. This reliability is supported by existing research: one study confirmed that meal frequency is a stable dietary indicator linked to BMI changes across age groups, while a systematic review noted FCVC sensitively reflects dietary-weight correlations (31, 32). Our low MAE values for NCP and FCVC align with these findings, verifying that these dietary attributes can be reliably captured for subsequent contribution analysis.

**Table 7:** MAE of RCA in each submodule.

Submodule	Attribute	MAE_mean_20
Submodule A	Height	0.0667
	Weight	0.0580
	NCP	0.0509
Submodule B	Age	0.0240
	Height	0.0582
	Weight	0.0514
Submodule C	Gender	0.0134
	Height	0.0628
	Weight	0.0465
	FCVC	0.0198
Average	0.0438	

Moreover, **Table 8** shows that besides physical indicators, FCVC contributes significantly to high-risk obesity stages (0.2564 for obesity I, 0.2319 for II, 0.2061 for III). This matches studies concluding vegetable intake is inversely associated with adiposity—especially in high-risk obesity—and that this correlation remains independent of demographic and lifestyle confounders (33, 34). Meanwhile, NCP plays a key role in distinguishing between underweight and normal dietary patterns (contributions 0.3538 and 0.3481). A prospective cohort study found abnormal meal frequency ties to BMI deviations (including underweight), and another study noted that regular meal frequency affects body composition regulation—both supporting NCP’s high contribution and reliability as a key feature (35, 36).

**Table 8:** The contribution belief matrix of obesity class.

Attribute Class label	Gender	Age	Height	Weight	FCVC	NCP
Insufficient	–	–	0.2630	0.3832	–	0.3538
Normal	–	–	0.2834	0.3685	–	0.3481
Overweight I	–	0.3973	0.3277	0.2750	–	–
Overweight II	–	0.4047	0.2761	0.3192	–	–
Obesity I	0.3848	–	0.2170	0.1418	0.2564	–
Obesity II	0.3876	–	0.2854	0.0951	0.2319	–
Obesity III	0.4154	–	0.2928	0.0858	0.2061	–

Furthermore, the contribution of physical indicators (height, weight) and demographic factors (age, gender) in **Table 8** is backed by epidemiological evidence. English cross-sectional surveys (1992–2011) found that BMI (from height/weight) has different obesity-discriminating effects in women and older adults, and gender differences impact severe obesity classification—matching height/weight’s core contribution and explaining gender’s 0.4154 contribution to obesity III (37). A UK birth cohort analysis also found that overweight progression mechanisms and age’s impact on overweight stages vary with age, especially in our 14–61-year-old sample—consistent with age’s differential contributions (0.3973 for overweight I, 0.4047 for II) (38).

These findings confirm that dietary behaviors, physical indicators, and demographic factors are sensitive to obesity risk, with their links to obesity verified by the literature. Thus, the DBRB-C model quantifies these factors via RCA and CBM, providing strong evidence for personalized dietary and lifestyle interventions to manage obesity risk.

### 3.3 Ablation study

As shown in **Table 9**, to systematically evaluate the contribution of each core component of the DBRB-C model to overall performance, a set of progressive comparison experiments was designed. Four model variants—BRB-A, BRB-B, BRB-C, and DBRB-C—were constructed to gradually analyze the performance gains of individual modules. The configurations are as follows:

**Table 9:** Performance comparison of model variants.

Model variant	Accuracy	Precision	Recall	F1 score
BRB-A	62.40%	75.04%	63.09%	60.34%
BRB-B	78.20% (↑15.80%)	78.78% (↑3.74%)	78.18% (↑15.09%)	78.23% (↑17.89%)
BRB-C	97.16% (↑18.96%)	97.14% (↑18.36%)	97.20% (↑19.02%)	97.16% (↑18.93%)
DBRB-C	98.58% (↑1.42%)	98.65% (↑1.51%)	98.54% (↑1.34%)	98.58% (↑1.42%)

- BRB-A: Baseline model, consisting of a single-layer BRB structure. Parameter optimization was performed using the DE algorithm alone.
- BRB-B: Based on BRB-A, PSO was introduced to form a DE-PSO hybrid optimization strategy, in order to validate the improvement of joint optimization on parameter search accuracy and model stability.
- BRB-C: DE optimization was retained, but a hierarchical inference architecture identical to that in DBRB-C was employed to assess the performance enhancement brought by the hierarchical structure.
- DBRB-C: The complete model integrating the hierarchical inference architecture, DE–PSO hybrid optimization, and RCA mechanism, aimed at comprehensively validating the overall performance advantage.

The experimental results show that BRB-A achieved an overall accuracy of 62.40% and an F1 score of 60.34%, indicating that the traditional single-layer BRB has significant limitations in multilevel obesity classification tasks. After introducing DE+PSO hybrid optimization, BRB-B achieved an accuracy of 78.20% and an F1 score of 78.23%, validating the effectiveness of the joint optimization strategy in enhancing parameter search precision and model stability. With the hierarchical inference structure, BRB-C achieved a leap in performance, with an accuracy of 81.36% and an F1 score of 79.27%. This indicates that the hierarchical architecture, through modular design, effectively mitigates the rule explosion problem, enhances the representation of obesity levels, and improves inference precision and efficiency. DBRB-C further incorporates the hybrid optimization strategy and RCA mechanism on top of BRB-C, resulting in an increase of 1.42% in both accuracy and F1 score, demonstrating the synergistic effect of the integrated modules.

### 3.4 Comparative experiment

To comprehensively evaluate the performance advantage of the proposed DBRB-C model in obesity-level prediction tasks, this study conducted systematic comparative experiments with mainstream machine learning classifiers. Meanwhile, a logistic regression model using only height and weight as input features (LR-HW) was additionally constructed to verify the source of the high accuracy of the DBRB-C model and ensure that its performance does not rely on a simple mapping of BMI calculated directly from height and weight. The evaluation metrics include accuracy, precision, recall, and F1 score. **Table 10** summarizes the overall performance results of the five models: CatBoost, random forest, conventional logistic regression, LR-HW, and DBRB-C.

**Table 10:** Performance comparison of classification models.

Dataset	Model variant	Accuracy	Precision	Recall	F1 score
Dataset 1	CatBoost (39)	94.10%	93.99%	93.96%	93.97%
	Random forest (40)	95.28%	94.00%	95.14%	94.51%
	Logistic regression (41)	87.94%	87.94%	87.90%	87.67%
	DBRB-C	98.58%	98.65%	98.54%	98.58%
	LR-HW	87.23%	87.62%	87.26%	87.10%
Dataset 2	CatBoost	82.30%	85.54%	78.98%	81.53%
	Random forest	85.09%	87.40%	84.75%	85.87%
	Logistic regression	73.91%	71.89%	70.33%	70.92%
	DBRB-C	97.31%	97.36%	97.31%	97.30%

Two datasets were involved in the experiment: dataset 1 includes samples from Colombia, Peru, and Mexico; dataset 2 includes samples from residents of Türkiye. To ensure fairness, comparability, and reproducibility of the comparative experiment, dataset 2 adopted the identical data processing and experimental design strategy as dataset 1. Stratified sampling was performed based on obesity-level labels; the training set, validation set, and test set were divided in a 6:2:2 ratio; and a fixed random seed was used to control the randomness of data partitioning.

**Figure 8:**
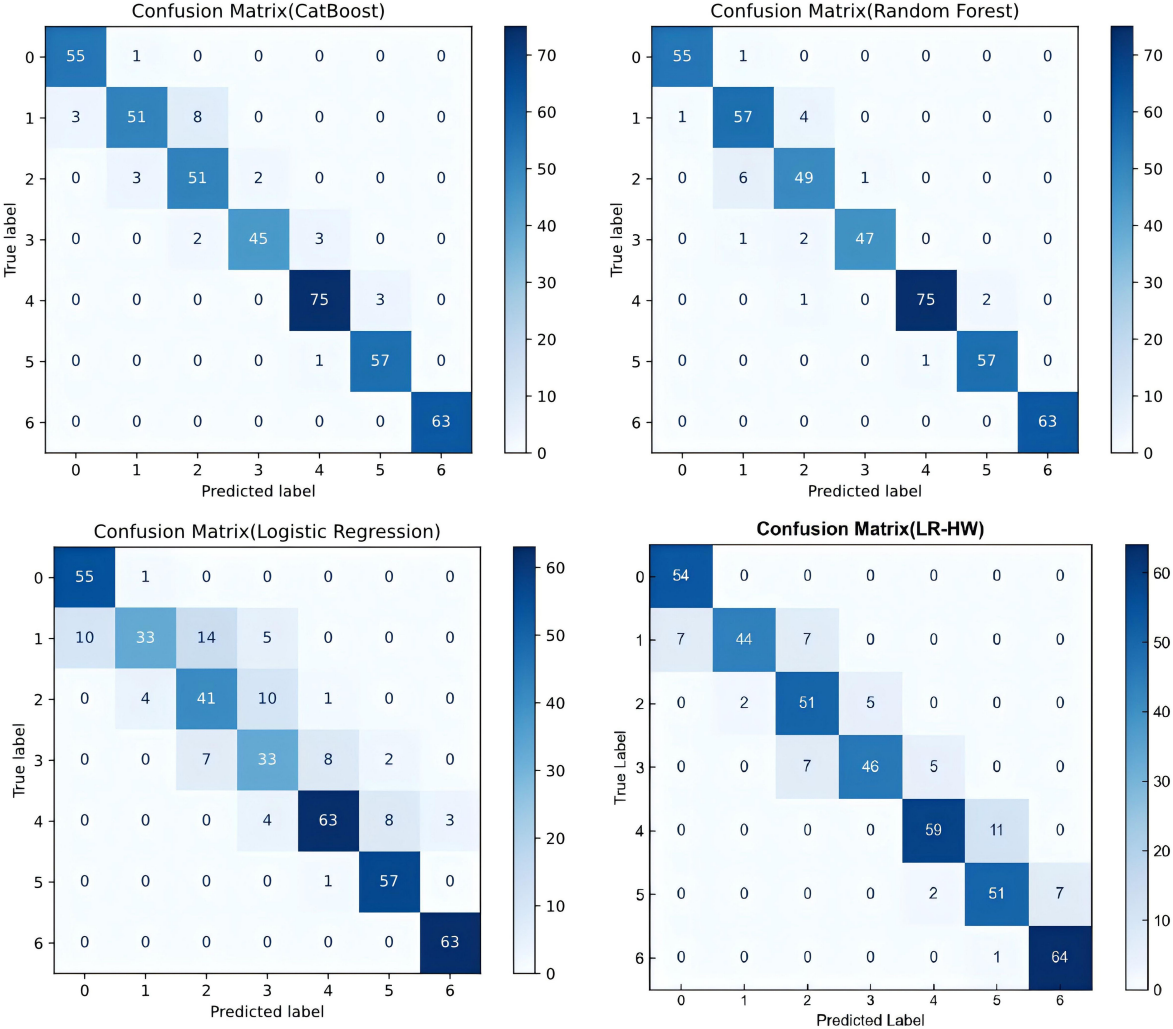
Confusion matrices of comparison models.

**Table 10** summarizes the overall performance results of the above five models on dataset 1 and dataset 2, while **Figure 8** presents the corresponding confusion matrix visualization results of each model on dataset 1, which can intuitively reflect the classification errors under different obesity levels.

Among them, CatBoost and random forest are ensemble learning models, conventional logistic regression and LR-HW are regression models, and DBRB-C is a hierarchical model based on a belief rule base. All models used exactly the same data samples without additional processing of the original data, so as to maximize the fairness of the comparative experiment and the objectivity and reliability of the results.

**Figure 9:**
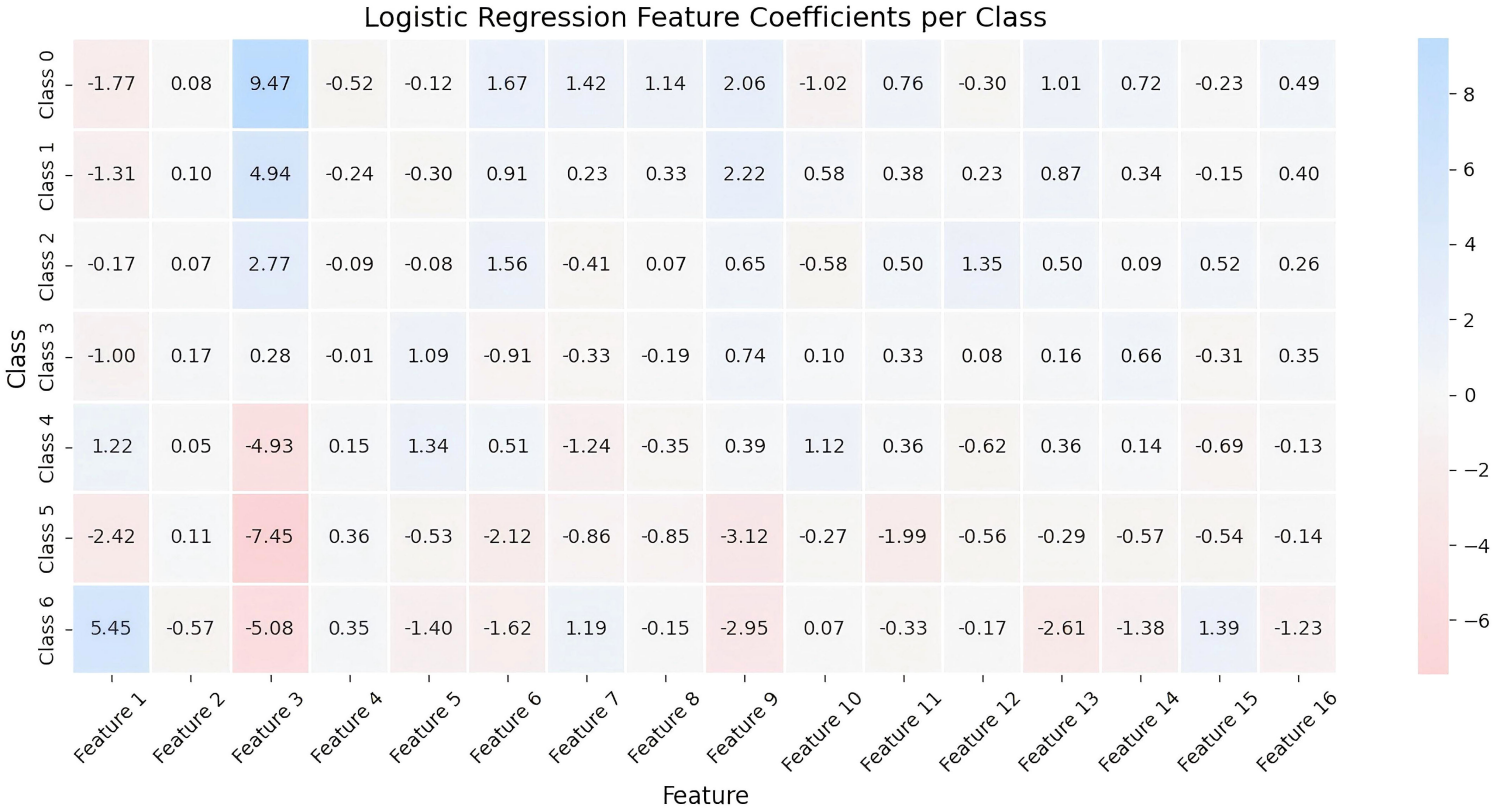
Logistic regression feature coefficients per class.

To comprehensively verify the performance integrity of DBRB-C in cross-population scenarios, in addition to the aforementioned classification metrics, key supplementary verification results were added for dataset 2: First, the reconstruction accuracy of DBRB-C for core features in dataset 2 was evaluated through the RCA mechanism, and the specific MAE values are presented in **Table 11**. Second, the contribution degree of each input feature to different obesity levels in dataset 2 was quantified using a contribution belief matrix, with detailed results shown in **Table 12**. The results of these two tables will be further analyzed in the “Discussion” section in combination with the model interpretability and cross-population applicability, so as to further confirm the reliability of DBRB-C in populations from different regions. Beyond performance metrics, interpretability is a key dimension for practical utility. Logistic Regression provides some linear interpretability through feature coefficients, which can be used to assess the influence of input variables on each class, as shown in **Figure 9**. However, it cannot capture the hierarchical dependencies among obesity levels, such as subtle differences between Obesity I and II. Furthermore, it lacks a structured reasoning path, limiting sample-level traceability.

**Figure 10:**
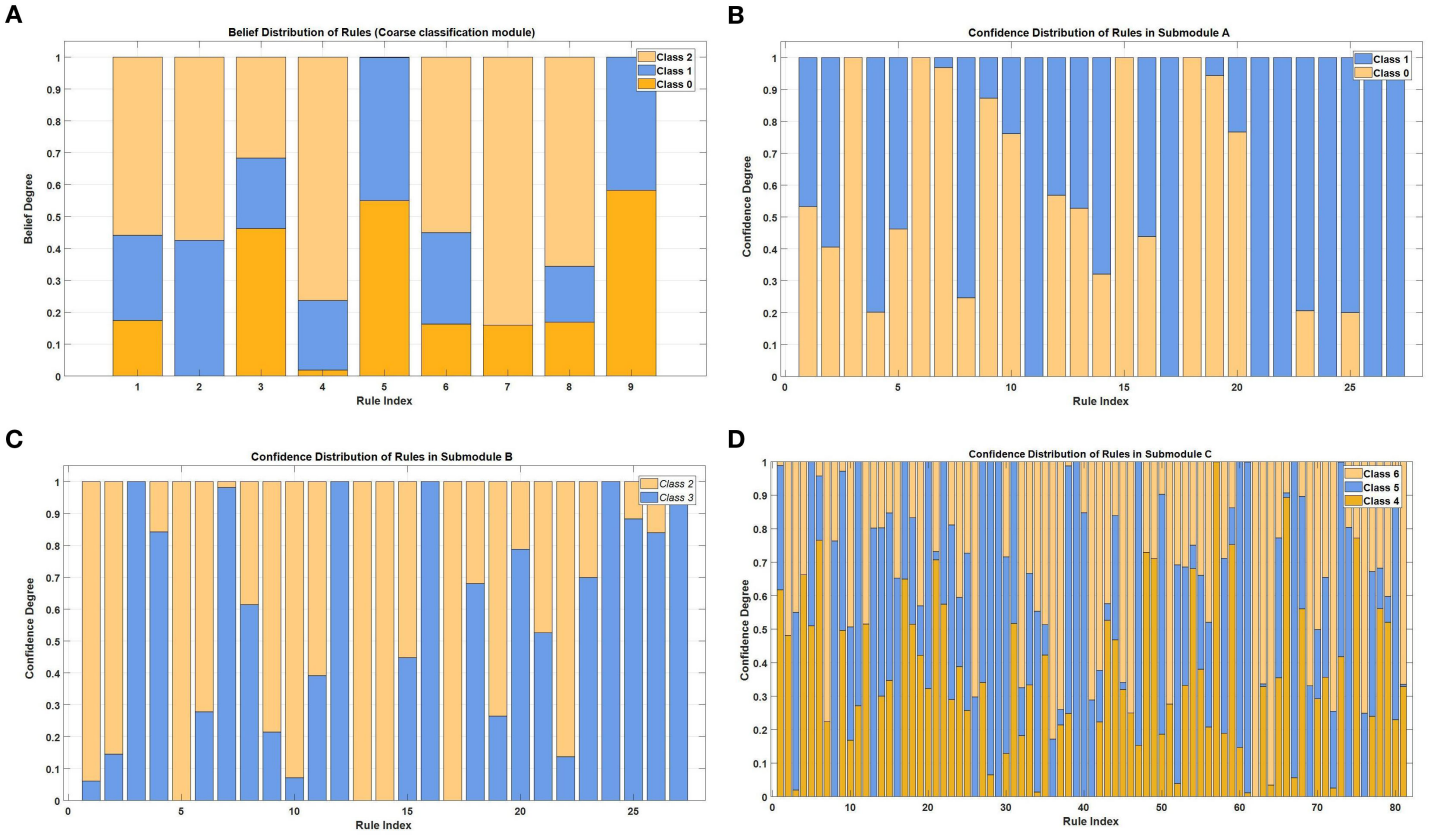
Confidence degree distributions of rules within each module of the DBRB-C hierarchical structure. **(A)** Coarse-classification layer (Lean, Overweight, Obese). **(B)** Submodule A (Insufficient Weight, Normal Weight). **(C)** Submodule B (Overweight I, Overweight II). **(D)** Submodule C (Obesity I, II, III).

By contrast, DBRB-C is composed of multiple rules, forming inherently hierarchical and transparent reasoning paths. The rule belief distributions of the coarse classification module and fine-grained submodules A, B, and C are shown in **Figure 10**. These distributions reflect the model’s rule usage preferences across different decision regions, enabling a decomposable and traceable decision process.

**Table 11:** MAE of RCA in each submodule (dataset 2).

Submodule	Attribute	MAE_mean_20
Submodule A	Frequency_of_Consuming_Vegetables	0.0277
	Calculation_of_Calorie_Intake	0.0379
	Physical_Excercise	0.0842
Submodule B	Overweight_Obese_Family	0.0597
	Frequency_of_Consuming_Vegetables	0.0331
	Number_of_Main_Meals_Daily	0.0569
Average	0.0499	

**Table 12:** The contribution belief matrix of obesity class (dataset 2).

Class label Attribute	Underweight	Normal	Overweight	Obesity
Overweight_Obese_Family	–	–	0.4057	0.3960
Frequency_of_Consuming_Vegetables	0.3311	0.2946	0.3614	0.3527
Number_of_Main_Meals_Daily	–	–	0.2329	0.2513
Calculation_of_Calorie_Intake	0.2092	0.2058	–	–
Physical_Excercise	0.4596	0.4996	–	–

## 4 Discussion

To improve the model’s generalization ability, this study used two cross-geographic and ethnic datasets, collected from Colombia, Peru, Mexico, and Turkey, denoted as dataset 1 and dataset 2, respectively. Both datasets adopted consistent feature classification logic, covering physiological-demographic, dietary, and lifestyle features, to ensure analytical comparability. Results showed that DBRB-C achieved accuracies of 98.58% and 87.23% on the two datasets, significantly outperforming mainstream models such as CatBoost and random forest, which verified the model’s generalization ability. Meanwhile, its hierarchical structure and joint feature selection strategy effectively alleviated the “rule explosion” problem, realizing a balance between performance and efficiency.

Feature analysis of dataset 1 showed that height and weight were the core bases for classification, and dietary features, including FCVC and NCP, played a key role in distinguishing high-risk obesity levels. To rule out the possibility that the model relied on simple BMI mapping calculated by height and weight, this study constructed a control model LR-HW with only height and weight as inputs. The accuracy of this model was 87.23%, 11.35 percentage points lower than that of DBRB-C, confirming that DBRB-C’s performance stemmed from the joint learning of multidimensional features such as physiology, diet, and lifestyle. Additionally, the reconstruction error of FCVC in dataset 1 was 0.0198, and that of NCP was 0.0509, both maintaining low levels. Their contribution to different obesity levels was comparable to that of height and weight, which verified the important role of dietary factors and provided a basis for personalized interventions.

To verify the cross-population reliability of the RCA mechanism, this study conducted supplementary analysis on dataset 2. Results showed that the reconstruction error of vegetable consumption frequency in the Turkish population was 0.0277, and that of calorie intake calculation habit was 0.0379. The low-error characteristics of these two indicators were highly consistent with the performance of FCVC and NCP in dataset 1. This result ruled out the possibility that low reconstruction error relied on a single dataset, confirming that the reconstruction reliability of the RCA mechanism had cross-population universality.

The contribution belief matrix in **Table 12** revealed the cross-population commonalities and specificities of obesity risk factors. In terms of commonalities, vegetable consumption frequency made significant contributions to all obesity levels in the Turkish population, with values ranging from 0.2946 to 0.3614, which echoed the contribution pattern of FCVC in dataset 1. Family history of overweight had the highest contributions to overweight and obesity in the Turkish population, at 0.4057 and 0.3960, respectively. This was consistent with the influence of gender on severe obesity in dataset 1, confirming that genetic and family factors were the core driving forces for obesity progression. In terms of specificities, physical exercise made prominent contributions to distinguishing between underweight and normal weight in the Turkish population, with values of 0.4596 and 0.4996, respectively. Although the contribution of the number of daily main meals to obesity was lower than that in dataset 1, it still maintained a positive correlation. These findings provided a basis for regionalized interventions.

In comparison, integrated models such as random forest performed moderately well on a single dataset, but they relied on complex tree structures and lacked hierarchical reasoning and contribution quantification mechanisms. As a result, they could not explain the classification basis, which limited their clinical application value. DBRB-C not only achieved optimal results in four core indicators, but also could provide regionalized intervention directions through the RCA mechanism and contribution matrix. For example, it suggested optimizing meal frequency for Latin American populations and strengthening the combined intervention of “exercise + vegetable intake” for Turkish populations. This was exactly the core disadvantage of “black-box models.”

In terms of interpretability, traditional logistic regression and LR-HW could only provide linear interpretation. They could not capture the hierarchical dependence among obesity subtypes, and LR-HW could not explain the role of modifiable factors such as diet and exercise at all. The rule-based structure of DBRB-C naturally formed a transparent reasoning path, and the rule belief distribution of its submodules could clearly trace decision preferences, which greatly improved the clinical practical value.

It should be noted that the model has limitations related to datasets. Longitudinal datasets are relatively scarce in public obesity research data, so this study used cross-sectional data, which can only capture static features and obesity status at specific time points and cannot track the dynamic changes of weight and diet over time. This makes the model perform well in static classification, but its prospective predictive ability, such as predicting “whether an individual will develop obesity in the next year,” has not been verified. In future research, incorporating 5–10 years of longitudinal tracking data can improve the accuracy of temporal prediction, clarify the causal relationship between factors, and further enhance the value of clinical prognosis assessment (42). Another limitation is the narrow range of features. Currently, the features are mainly anthropometric and dietary indicators, without fully integrating inflammatory indicators such as C-reactive protein, metabolic markers such as insulin resistance index, and genomic data. This may affect the ability to predict early risks of obesity-related complications such as type 2 diabetes.

## 5 Conclusions

In conclusion, the DBRB-C model represents a novel and effective approach for multilevel obesity classification and risk assessment. The model’s hierarchical architecture, combined with a joint feature selection strategy and the DE+PSO hybrid optimization, has demonstrated significant improvements in predictive accuracy, computational efficiency, and interpretability, as evidenced by our experimental results. Key findings highlight the critical contributions of anthropometric measures, dietary behaviors such as NCP and FCVC, and demographic factors, including gender and age, in determining obesity risk. By integrating these features into a hierarchical and modular framework, the DBRB-C model not only achieves high classification performance but also provides traceable and quantifiable insights into the relative contributions of obesity-influencing factors. This capability facilitates personalized dietary and lifestyle interventions, enhancing the model’s practical utility in clinical and public health settings. Furthermore, the RCA mechanism allows for precise reconstruction of input features, ensuring reliable interpretation of predictive outcomes and supporting evidence-based decision-making. Overall, the DBRB-C model exemplifies the potential of combining interpretable AI with structured medical knowledge, offering a promising direction for precision nutrition, obesity management, and preventive health strategies. Its robust performance and transparent reasoning lay the foundation for more personalized and effective interventions, ultimately contributing to improved health outcomes and informed public health planning.

## Data Availability

The datasets presented in this study can be found in online repositories. The names of the repository/repositories and accession number(s) can be found below: https://archive.ics.uci.edu/dataset/544/estimation+of+obesity+levels+based+on+eating+habits+and+physical+conditionhttps://www.kaggle.com/datasets/suleymansulak/obesity-dataset.
